# Outcome nach Gelenkersatz bei Patienten mit rheumatoider Grunderkrankung

**DOI:** 10.1007/s00393-023-01424-4

**Published:** 2023-10-04

**Authors:** Dominik Emanuel Holzapfel, Max Thieme, Tobias Kappenschneider, Sabrina Holzapfel, Günther Maderbacher, Markus Weber, Joachim Grifka, Matthias Meyer

**Affiliations:** 1 Medical Center, Department of Orthopaedic Surgery Asklepios Klinikum Bad Abbach, Regensburg University, Kaiser-Karl V.-Allee 3, 93077 Bad Abbach, Deutschland; 2 Center of Oncology, Hospital Barmherzige Brüder, Medical Center, Regensburg, Deutschland; 3 Department of Neonatology, Children’s Hospital St. Hedwig Barmherzige Brüder, Medical Center, Regensburg, Deutschland

**Keywords:** Total joint replacement, Postoperative Komplikationen, Patient-reported outcome, Hüfttotalendoprothesenimplantation, Knietotalendoprothesenimplantation, Total joint replacement, Postoperative complications, Patient-reported outcome, Total hip arthroplasty, Total knee arthroplasty

## Abstract

**Hintergrund:**

Der künstliche Gelenkersatz ist für Patienten mit fortgeschrittenen rheumatisch degenerativen Gelenkveränderungen eine sinnvolle Therapieoption. Ziel dieser Studie war es, den Einfluss rheumatischer Grunderkrankungen auf postoperative Komplikationen und „patient-reported outcome“ (PRO) nach elektivem Gelenkersatz („total joint replacement“ [TJR]) zu untersuchen.

**Materialien und Methoden:**

In einer retrospektiven Analyse von 9149 Patienten nach elektiver Knie- oder Hüfttotalendoprothesenimplantation (TKR und THR) wurden Komplikationsraten und PRO von Patienten mit und ohne rheumatische Grunderkrankung („rheumatic disease“ [RD]) verglichen. Multivariate logistische Regressionsmodelle wurden verwendet, um festzustellen, ob rheumatische Grunderkrankungen einen unabhängigen Risikofaktor für verschiedene Komplikationen darstellen.

**Ergebnisse:**

RD-Patienten hatten nach TJR in den univariaten Analysen ein erhöhtes Risiko für internistische Komplikationen (7,1 % vs. 5,2 %, *p* = 0,028) und Clavien-Dindo-Grad-IV-Komplikationen (2,8 % vs. 1,8 %, *p* = 0,048). Dies konnte in multivariaten statistischen Analysen bestätigt werden (*p* = 0,034). Die Raten für operative Revisionen und chirurgische Komplikationen waren vergleichbar (2,5 % vs. 2,4 %, *p* = 0,485). Die Analyse des PRO ergab eine höhere Responderrate bei Patienten mit RD nach TKR (91,9 % vs. 84,5 %, *p* = 0,039). Die Responderrate nach THR war hingegen vergleichbar (93,4 % vs. 93,2 %, *p* = 0,584).

**Schlussfolgerung:**

Trotz teilweise erhöhter postoperativer Komplikationsraten zeigen Patienten mit rheumatischer Grunderkrankung 1 Jahr nach Operation ein vergleichbares Outcome nach TJR. RD-Patienten nach TKR zeigen sogar höhere Responderraten. RD-Patienten sind zwar eine vulnerable Patientengruppe, können aber dennoch von einem Gelenkersatz profitieren.

**Zusatzmaterial online:**

Die Online-Version dieses Beitrags (10.1007/s00393-023-01424-4) enthält Tab. S1.

## Abstract (en)

**Background:**

Artificial joint replacement is a meaningful treatment option for patients with advanced rheumatic degenerative joint diseases. The aim of this study was to investigate the influence of the underlying rheumatic diseases on postoperative complications and patient-reported outcome (PRO) after elective total joint replacement (TJR).

**Material and methods:**

In a retrospective analysis of 9149 patients with elective total knee or total hip arthroplasty (TKR and THR), complication rates and PRO of patients with and without rheumatic diseases (RD) were compared. Multivariate logistic regression models were used to determine whether the underlying rheumatic disease was an independent risk factor for various complications.

**Results:**

In the univariate analyses the RD patients had an increased risk of medical complications (7.1% vs. 5.2%; *p* = 0.028) and Clavien-Dindo grade IV complications (2.8% vs. 1.8%; *p* = 0.048) after TJR. This was confirmed in multivariate statistical analyses (*p* < 0.034). The rates for operative revisions and surgical complications were comparable (2.5% vs. 2.4%; *p* = 0.485). Analysis of the PRO showed a higher responder rate in patients with RD after TKR (91.9% vs. 84.5%, *p* = 0.039). In contrast, the responder rate in patients with RD after THR was comparable (93.4% vs. 93.2%, *p* = 0.584).

**Conclusion:**

Despite increased postoperative complication rates, patients with underlying rheumatic diseases showed a comparable outcome 1 year after TJR. After TKR the RD patients showed even higher responder rates. Although RD patients are a vulnerable patient group, they can still benefit from joint replacement.

## Hintergrund

Die rheumatoide Arthritis ist eine multifaktorielle, inflammatorische Autoimmunerkrankung mit einer globalen Inzidenz von etwa 0,5–1 % [1, 2]. Unbehandelt führen die entzündlichen Prozesse primär zu einer fortschreitenden Destruktion der kleinen Gelenke und schließlich auch der großen Gelenke wie Knie und Hüfte [3]. Seit der Optimierung der medikamentösen Therapie mithilfe von „disease-modifying antirheumatic drugs“ (DMARDs) und anderen „biologic immunomodulating agents“ ist zwar ein Rückgang notwendiger operativer Versorgungen zu beobachten, bei fortgeschrittenen, symptomatischen Gelenkveränderungen ist ein „total joint replacement“ [TJR]) aber weiterhin eine verbreitete Therapieoption [4, 5]. Das Lebenszeitrisiko eines Patienten mit rheumatischer Grunderkrankung (RD) für ein TJR liegt etwa zwischen 17 und 25 %. Dabei ist das Ziel der Operation Schmerzen zu lindern und die Funktion des betroffenen Gelenkes zu verbessern [4, 6]. Es wird angenommen, dass RD-Patienten eine höhere Komplikationsrate nach TJR aufweisen. Mögliche Gründe sind der entzündliche Charakter der Grunderkrankung, das Nebenwirkungsprofil der medikamentösen Therapie, der Grad präoperativer rheumatischer Deformität und Destruktion oder auch Kombinationen [7]. Diskutabel ist nun, ob RD-Patienten von einem TJR profitieren oder die Nachteile überwiegen. Ziel unserer Studiengruppe war es, in einem retrospektiven Setting das eigene Patientengut bezüglich eines möglichen Zusammenhangs zwischen rheumatischen Grunderkrankungen, postoperativen Komplikationen und „patient-reported outcome“ (PRO) zu analysieren. Unsere Hypothese war, dass RD-Patienten höhere postoperative Komplikations- und Revisionsraten nach Knie- oder Hüftgelenkersatz haben und ein schlechteres PRO zeigen als Patienten ohne rheumatische Grunderkrankung.

## Material und Methoden

Über das Krankenhausinformationssystem unserer hausinternen Datenbank (ORBIS, Agfa healthcare, Florenz, Italien) konnten über einen Zeitraum von Juni 2011 bis Dezember 2019 in einem retrospektiven Setting Daten von 9149 Patienten ermittelt werden, die im Rahmen eines stationären Aufenthaltes in unserer orthopädischen Universitätsklinik eine künstliche Hüft- oder Kniegelenkersatzoperation erhalten hatten.

Über zugeordnete Fallidentifikationsnummern, ICD-10(International Statistical Classification of Diseases and Related Health Problems)-Codes und OPS(Operation and Procedure)-Codes konnten schließlich aus diesem Patientengut diejenigen Patienten identifiziert werden, die an einer RD erkrankt sind (*n* = 603). Darunter wurden Patienten mit rheumatoider Arthritis (RA), Psoriasisarthritis (PsA), Spondylarthritis (SpA) und Kollagenosen zusammengefasst.

Des Weiteren wurden unter anderem soziodemografische Merkmale und andere Daten wie Alter, Geschlecht, ASA(American Society of Anesthesiologists)-Score, Operationsdauer, Hospital Frailty Risk Score (HFRS), internistische und chirurgische Komplikationen sowie operative Revisionsraten < 60 Tage erfasst.

Aufgrund der insgesamt geringen Anzahl fassten wir die einzelnen Komplikationen in der Analyse unter internistische und chirurgische Komplikationen zusammen. Internistische Komplikationen enthielten kardiologische Komplikationen (Myokardinfarkte, Herzrhythmusstörungen), pulmonale Komplikationen (Pneumonien, Lungenödeme), renale Komplikationen (Niereninsuffizienz, Elektrolytentgleisungen). Chirurgische Komplikationen beinhalteten Frakturen, Wundheilungsstörungen und mechanische Komplikationen.

Die Erhebung des HFRS erfolgte mit einer Zuordnung von ICD-10-Codes zu den jeweiligen Patienten. Dabei sind 109 ICD-10-Codes charakteristisch für „Frailty“ und werden dem jeweiligen „Frailty“-Schweregrad in Form von Punktewerten gemäß Definition nach Gilbert et al. [8] zugeordnet. Je nach Summenscore wird das Patientengut dann in 3 Gruppen eingeteilt. Die Klassifikation erfolgt in ein geringes (HFRS < 5), mittleres (HFRS zwischen 5 und 15) und hohes „Frailty“-Risiko (HFRS > 15). Die Summe des maximal erreichbaren Scores beträgt 173,2 Punkte [5]. Außerdem wurden die Konsequenzen der erfassten Komplikationen im Rahmen der Clavien-Dindo-Klassifikation [9] erfasst. Dabei wird die jeweilige Therapie, angepasst an den Schweregrad einer Komplikation, in eine von 5 Stufen kategorisiert. Stufe IV hat eine intensivmedizinische Therapie zur Folge und ist lebensbedrohlich. Diese Stufe IV nach Clavien-Dindo wurde während des stationären Krankenhausaufenthalts in unserer Datenbank erfasst.

Aus dem hausinternen Gelenkregister konnten „patient-reported outcome measurements“ (PROMs) herausgefiltert werden. Diese umfassten den Western Ontario and McMaster Universities Arthritis Index (WOMAC) [10] und den European Quality of Life 5 Dimensions(EQ-5D)-Fragebogen [11]. Der WOMAC und der EQ-5D wurden präoperativ und 1 Jahr postoperativ erhoben. Die Daten der beiden Fragebogeninstrumente waren jeweils nur für eine Subgruppe der Studienpopulation verfügbar. Grund dafür ist, dass die Datenerfassung der PROMs in unserer Klinik erst mit der Implementierung eines zertifizierten Endoprothesenzentrums im Oktober 2012 aufgenommen wurde und einige Patienten für die Follow-up-Erhebung nicht mehr zur Verfügung standen.

Um einen statistisch definierten Effekt zwischen Respondern und Non-Respondern bei RA-Patienten und Nicht-RA-Patienten nach künstlichem Gelenkersatz messen zu können, wurden die Outcome Measures in Rheumatology and Osteoarthritis Research Society International Consensus-Kriterien (OMERACT-OARSI) in unserer Auswertung berücksichtigt [12].

Die statistische Auswertung erfolgte mit der Statistiksoftware SPSS 26 (IBM, Armonk, New York, United States). Kontinuierliche Daten wurden als Mittelwert (Standardabweichung) angegeben. Gruppenvergleiche wurden mit zweiseitigen t‑Tests durchgeführt. Kategoriale Daten wurden in absoluten und relativen Häufigkeiten angegeben und mittels Pearson-Chi-Quadrat-Test zwischen den Gruppen verglichen. Das Signifikanzniveau aller Analysen wurde als *p*-Wert < 0,05 definiert. Um Störgrößen auszuschließen, wurden multivariate Analysen mittels logistischer Regressionsanalysen berechnet. Allgemein bekannte berücksichtigte Einflussgrößen waren dabei Alter, Geschlecht, Art der Operation, „Frailty“, Operationsdauer in Minuten [13]. Alle TJR-Operationen wurden von zertifizierten Hauptoperateuren an einer orthopädischen Universitätsklinik (Endoprothesenzentrum der Maximalversorgung) durchgeführt.

## Ergebnisse

Im Beobachtungszeitraum erhielten 9149 Patienten einen Hüft- oder Kniegelenkersatz. Bei 6,6 % (603/9149) der Patienten lag eine rheumatologische Grunderkrankung vor. Dabei lag bei 82,3 % eine rheumatoide Arthritis (RA), bei 0,3 % eine Psoriasisarthritis (PsA), bei 4,0 % eine Spondylarthritis (SpA) und bei 13,4 % eine Kollagenose als Grunderkrankung vor. Die demografischen Daten der Studiengruppe sind in Tab. 1 ersichtlich.

**Table 1:** 

TJR	Gesamtpopulation (*n* = 9149) %	RD-Patienten (*n* = 603) %	Nicht-RD-Patienten (*n* = 8546) %	*p*-Wert
Frauen	57,7 % (*n* = 5275)	72,5 % (*n* = 437)	56,6 % (*n* = 4838)	*<* *0,001*
Männer	42,3 % (*n* = 3874)	27,5 % (*n* = 166)	43,4 % (*n* = 3708)	*<* *0,001*
THR	55,1 % (*n* = 5045)	44,8 % (*n* = 270)	55,9 % (*n* = 4775)	*<* *0,001*
TKR	44,9 % (*n* = 4104)	55,2 % (*n* = 333)	44,1 % (*n* = 3771)	*<* *0,001*
Alter (Jahre) Mw (SD)	66,3 (10,8)	65,7 (11,0)	66,3 (10,8)	0,186
ASA-Score Mw (SD)	2,2 (0,6)	2,4 (0,5)	2,2 (0,6)	*<* *0,001*
HFRS Mw (SD)	1,1 (1,9)	1,3 (1,8)	1,1 (1,9)	*0,012*
Stationärer Aufenthalt (Tage) Mw (SD)	9,0 (4,0)	9,7 (4,3)	9,1 (4,2)	*0,004*
Operationsdauer (min) Mw (SD)	82 (14)	80 (31)	82 (14)	0,458

*TJR* „total joint replacement“, *RD* „rheumatic disease“, *THR* „total hip replacement“, *TKR* „total knee replacement“, *Mw* Mittelwert, *SD* Standardabweichung, *ASA* American Society of Anesthesiologists, *HFRS* Hospital Frailty Risk Score, signifikante Werte (*p*-Wert < 0,05) sind kursiv abgebildet

**Figure 1:**
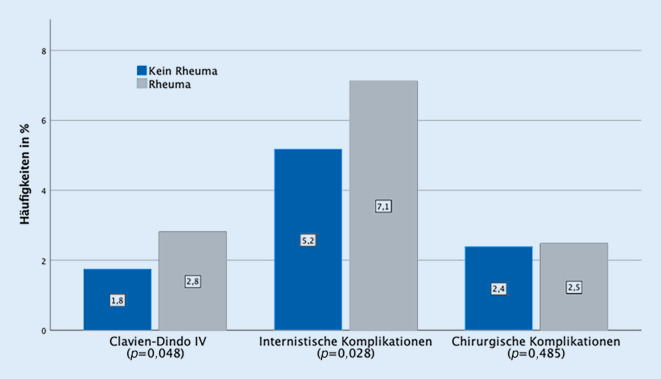

Unter den Patienten mit rheumatischer Grunderkrankung waren mehr Frauen, die einen künstlichen Kniegelenkersatz erhalten haben (Tab. 1). Unter den RD-Patienten war der ASA-Score signifikant höher als bei anderen TJR-Patienten. RD-Patienten erlitten häufiger internistische Komplikationen und verlegungswürdige Komplikationen (Clavien-Dindo IV) als Patienten ohne rheumatische Grunderkrankung (Tab. 2). Dies gilt für TJRs insgesamt (internistische Komplikation *p* < 0,028, Clavien-Dindo IV *p* < 0,048) und für alle THRs („total hip replacement“) (internistische Komplikation *p* < 0,036, Clavien-Dindo IV *p* < 0,01) (Tab. 2 und Abb. 1). Bei den TKRs („total knee replacement“) gab es keine signifikanten univariaten Zusammenhänge mit internistischen Komplikationen oder Komplikationen Clavien-Dindo IV, es konnte aber eine signifikant höhere Korrelation mit Revisionsoperationen < 60 Tage beobachtet werden (*p* < 0,045) (Tab. 2).

**Table 2:** 

**„Total joint replacement“ (TJR)**				
*TJR*	*RD-Patienten* *n* = 603 % (*n*)	*Nicht-RD-Patienten* *n* = 8546 % (*n*)	*Total* *n* = 9149 % (*n*)	*p‑Wert*
Revisionsoperation < 60 Tage	6,3 % (38)	5,0 % (431)	5,1 % (469)	0,106
Internistische Komplikationen	7,1 % (43)	5,2 % (443)	5,3 % (486)	*0,028*
Chirurgische Komplikationen	2,5 % (15)	2,4 % (205)	2,4 % (220)	0,485
Clavien-Dindo IV	2,8 % (17)	1,8 % (150)	1,8 % (167)	*0,048*
**„Total hip replacement“ (THR)**				
*THR*	*RD-Patienten* *n* = 270 % (*n*)	*Nicht-RD-Patienten* *n* = 4775 % (*n*)	*Total* *n* = 5045 % (*n*)	*p‑Wert*
Revisionsoperation < 60 Tage	5,2 % (14)	5,2 % (248)	5,2 % (262)	0,568
Internistische Komplikationen	7,4 % (20)	4,7 % (224)	4,8 % (244)	*0,036*
Chirurgische Komplikationen	3,0 % (8)	2,9 % (138)	2,9 % (146)	0,526
Clavien-Dindo IV	4,1 % (11)	1,7 % (82)	1,8 % (93)	*0,010*
**„Total knee replacement“ (TKR)**				
*TKR*	*RD-Patienten* *n* = 333 % (*n*)	*Nicht-RD-Patienten* *n* = 3771 % (*n*)	*Total* *n* = 4104 % (*n*)	*p‑Wert*
Revisionsoperation < 60 Tage	7,2 % (24)	4,9 % (183)	5,0 % (207)	*0,045*
Internistische Komplikationen	6,9 % (23)	5,8 % (219)	5,9 % (242)	0,239
Chirurgische Komplikationen	2,1 % (7)	1,8 % (67)	1,8 % (74)	0,394
Clavien-Dindo IV	1,8 % (6)	1,8 % (68)	1,8 % (74)	0,606

*RD* „rheumatic disease“

In den multivariaten Analysen bestätigte sich, dass RD nach TJR ein unabhängiger Risikofaktor für internistische Komplikationen (OR [Odds Ratio] 1,48, Konfidenzintervall [KI] 1,03–2,13, *p* = 0,034) und intensivpflichtige Komplikationen (Clavien-Dindo IV) (OR 1,70, KI 1,00–2,90, *p* = 0,049) ist. Dabei wurden die Variablen Alter, Geschlecht, Art der Operation, „Frailty“ und Operationsdauer in Minuten als Risikofaktoren berücksichtigt. Innerhalb der Kovariablen zeigten die Parameter höheres Alter (OR 1,05, KI 1,03–1,06, *p* < 0,001), Art der Operation (OR 0,79, KI 0,64–0,97, *p* = 0,026) und „Frailty“ (OR 1,55, KI 1,49–1,61, *p* < 0,001) ebenfalls einen unabhängigen Einfluss auf internistische Komplikationen. Bezüglich intensivpflichtiger Komplikationen zeigten die Kovariablen Alter (OR 1,06, KI 1,04–1,08, *p* < 0,001) und HFRS (OR 1,12, KI 1,06–1,18, *p* < 0,001) einen unabhängigen Effekt auf die Wahrscheinlichkeit ihres Auftretens (Tab. 3).

**Table 3:** 

TJR	OR (95 %-KI)	*p*-Wert
*Internistische Komplikationen*		
RD	1,48 (1,03–2,13)	*0,034*
Alter	1,05 (1,03–1,06)	*<* *0,001*
Geschlecht (♀)	0,90 (0,72–1,12)	0,35
Operationsart (THR)	0,79 (0,64–0,97)	*0,026*
HFRS	1,55 (1,49–1,61)	*<* *0,001*
Operationsdauer	1,00 (1,00–1,00)	0,997
*Clavien-Dindo IV*		
RD	1,70 (1,00–2,90)	*0,049*
Alter	1,06 (1,04–1,08)	*<* *0,001*
Geschlecht (♀)	1,04 (0,74–1,46)	0,826
Operationsart (THR)	1,14 (0,82–1,58)	0,443
HFRS	1,12 (1,06–1,18)	*<* *0,001*
Operationsdauer	1,00 (1,00–1,00)	0,364

*TJR* „total joint replacement“, *OR* Odds Ratio, *KI* Konfidenzintervall, *RD* „rheumatic disease“, THR „total hip replacement“, *HFRS* Hospital Frailty Risk Score, signifikante Werte (*p*-Wert < 0,05) sind kursiv abgebildet

In einer Subgruppenanalyse des WOMAC-Index von 2705 Patienten nach TJR gemäß den OMERACT-OARSI-Kriterien zeigte sich in der Gesamtgruppe eine Responderrate von 90,2 % (2440/2705). Der Anteil an Respondern nach THR war in der Gesamtpopulation höher als nach TKR (93,5 % (1451/1552) vs. 85,8 % (989/1153)).

**Figure 2:**
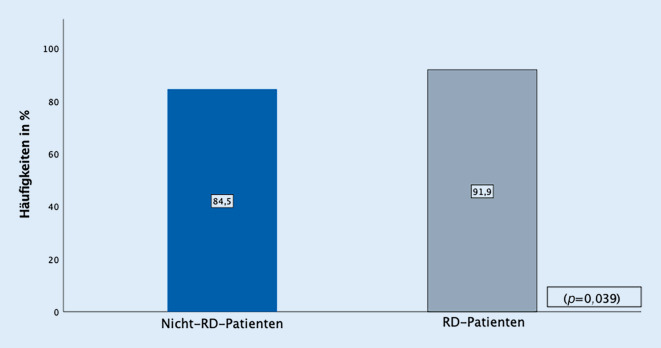

Im Vergleich von Patienten mit und ohne rheumatische Grunderkrankungen zeigten sich im Gesamtkollektiv (RD-Patienten 92,6 % vs. Nicht-RD-Patienten 89,5 %, *p* = 0,131) und im THR-Kollektiv vergleichbare Responderraten (RD-Patienten 93,4 % vs. Nicht-RD-Patienten 93,2 %, *p* = 0,584). Im TKR-Kollektiv zeigten RD-Patienten jedoch eine signifikant höhere Responderrate als Nicht-RD-Patienten (91,9 % vs. 84,5 %, *p* = 0,039) (Abb. 2).

Die Auswertung des EQ-5D zeigte, dass RD-Patienten vor und nach TJRs eine signifikant geringere allgemeine gesundheitsbezogene Lebensqualität haben als Nicht-RA-Patienten (s. Anhang Appendix 1).

## Diskussion

Bei der Betrachtung der demografischen Daten waren die Geschlechterverteilung und die Verteilung der Operationsart (TKR/THR) in der Gesamtpopulation relativ ausgeglichen. In der Gruppe der RD-Patienten zeigte sich mit 72,5 % (*n* = 437) ein deutlich höherer Frauenanteil. Dies deckt sich weitgehend mit den Erkenntnissen aus anderen Studien, die einen 2‑ bis 3‑mal höheren Anteil weiblicher Rheumapatienten beschreiben [3, 14].

Zusammenfassend zeigte die Analyse ein erhöhtes Risiko für internistische Komplikationen und intensivpflichtige Komplikationen für RD-Patienten nach TJR. Ähnliche Ergebnisse finden sich auch in der Literatur. Lian et al. beobachteten nach TJR ebenso erhöhte internistische Komplikationen [15]. Gründe dafür sind unter anderem auch das vulnerable Patientengut [16] und die hohe Komorbiditätsrate [2] bei RD-Patienten. Auch in der vorliegenden Datenauswertung kann anhand signifikant höherer Mittelwerte des ASA-Scores (2,4 [0,5] vs. 2,2 [0,6]; *p*-Wert < 0,001) beobachtet werden, dass RD-Patienten kränker zu sein scheinen.

Ein hohes Risiko für chirurgische Komplikationen nach TJR bei RD-Patienten konnte in zahlreichen Studien nachgewiesen werden [5, 17–19]. Zang et al. konnten in einer Metaanalyse von 23 Studien beispielsweise ein höheres Risiko für Hüftluxationen, periprothetische Infektionen, postoperative Wundinfektionen und Revisionsoperationen zeigen [5]. In unserer Studiengruppe konnte entgegen der initialen Hypothese in der Gesamtpopulation keine signifikante Korrelation von chirurgischen Komplikationen oder Revisionsoperationen nach TJR bei RD-Patienten gefunden werden. Sie waren aber zumindest vergleichbar. Lediglich die RD-Patienten nach künstlichem Kniegelenkersatz zeigten signifikant höhere operative Revisionsraten. In der Literatur werden diesbezüglich auch aufgrund der Verwendung von DMARDs und Biologika erhöhte Infektraten und Wundheilungsstörungen beschrieben [20–22].

Für RD-Patienten nach TJR besteht ein erhöhtes Risiko für internistische und intensivpflichtige Komplikationen

In der Subgruppenanalyse des WOMAC-Index konnten bezüglich der Gesamtresponder nach OMERACT-OARSI (THR 93,5 % Responder; TKR 85,8 % Responder) ähnliche Daten beobachtet werden wie in der Literatur (Escobar et al.: THR 95 % Responder; TKR 86 % Responder) [23].

Zudem konnte nachgewiesen werden, dass RD-Patienten nach TKR eine signifikant höhere Responderrate zeigen als Nicht-RD-Patienten. Nach THR ergaben sich bei RD-Patienten und Nicht-RD-Patienten vergleichbare Responder-Raten. Hawker et al. konnten jedoch zeigen, dass die Wahrscheinlichkeit für ein gutes Outcome mit einem schlechteren präoperativen WOMAC-Summenscore steigt [24].

Burn et al. erörterten, dass die Lebensqualität für RD-Patienten nach TJR ebenso deutlich verbessert werden kann [17]. Unsere Auswertung des EQ-5D zeigen, dass RD-Patienten postoperativ zwar eine Verbesserung der allgemeinen gesundheitsbezogenen Lebensqualität haben, diese ist aber prä- und postoperativ bei RD-Patienten signifikant geringer als bei Nicht-RD-Patienten. Dies scheint eine logische Konsequenz aus den Gegebenheiten der rheumatoiden Grunderkrankung zu sein. Postoperativ besserte sich die allgemeine gesundheitsbezogene Lebensqualität jedenfalls in beiden Gruppen signifikant.

Hauptlimitation der Studie ist das retrospektive Design. Die vorliegenden Daten können nicht die Qualität und Vollständigkeit prospektiv erhobener Daten haben. Bei der Datenerhebung über OPS-Codes und Fallzahlen kann eine Fehleranfälligkeit bei der Auswertung nicht hundertprozentig ausgeschlossen werden. Auch die Informationsfülle ist bei einer hausinternen Datenbank begrenzt. Andere Parameter mit Einfluss auf das postoperative Outcome wie beispielsweise Body Mass Index oder sozioökonomischer Status waren nicht enthalten und konnten folglich nicht einbezogen werden. Weiterhin standen keine Informationen zu Art, Schweregrad und Therapie der rheumatologischen Grunderkrankung zu Verfügung. Bei der Auswertung der PRO-Datensätze wäre ein längerer Nachbeobachtungszeitraum zur Beurteilung eines langfristigen Outcomes von Vorteil. Stärke der Studie ist die hohe Anzahl an Patienten mit rheumatologischer Grunderkrankung und Gelenkersatz in einem elektiven Setting mit standardisierten Behandlungsabläufen und Nachbehandlungsschemata. Prospektive randomisierte und multizentrische Studien mit Langzeit-Follow-up können ein Ansatz sein, um weitere und bessere Erkenntnisse zu erbringen.

## Fazit für die Praxis

- Patienten mit rheumatologischer Grunderkrankung (RD) haben nach elektivem Gelenkersatz ein erhöhtes Komplikationsrisiko. Dennoch profitieren RD-Patienten in Bezug auf das „patient-reported outcome“ tendenziell sogar mehr von einem Gelenkersatz als Patienten ohne rheumatologische Grunderkrankung.
- Rheumapatienten erleiden postoperativ häufiger internistische Komplikationen.
- Die Revisionsrate ist bei Rheumapatienten nach Kniegelenkersatz erhöht.
- Rheumapatienten haben eine geringere gesundheitsbezogene Lebensqualität vor und nach TJR („total joint replacement“).
- Rheumapatienten profitieren von einem Gelenkersatz mindestens genauso viel wie Patienten ohne rheumatologische Grunderkrankung.

### Supplementary Information




