# Supplementary material for: Outcome of joint replacement in patients with underlying rheumatoid disease
Source: Z Rheumatol. 2023 Oct 4;82(10):825–33. [Article in German] doi: 10.1007/s00393-023-01424-4 (PMC10695889; doi:10.1007/s00393-023-01424-4)
Supplement: Supplementary file 1 [file 393_2023_1424_MOESM1_ESM.docx]

**Tab. S1: Auswertung des prä- und postoperativen WOMAC-Index und des EQ-5D bei RA- und Nicht-RA-Patienten nach TJR, THR und TKR**

| **Total Joint Replacements** | | | | |
| --- | --- | --- | --- | --- |
| **TJRs** | **Rheumatic disease**  n =  mean SD) | **No rheumatic disease**  n =  mean SD) |  | **p-value** |
| **WOMAC Prä gesamt** | 60.8 (16.8) | 58.3 (16.3) |  | **.009** |
| **WOMAC Prä Schmerz** | 13.3 (3.8) | 12.4 (3.7) |  | **<.001** |
| **WOMAC Prä Steifheit** | 4.8 (1.9) | 4.9 (1.8) |  | .109 |
| **WOMAC Prä Funktion** | 42.9 (12.9) | 41.0 (12.3) |  | **.010** |
| **WOMAC Post gesamt** | 20.5 (18.8) | 20.0 (18.7) |  | .710 |
| **WOMAC Post Schmerz** | 3.9 (4.0) | 3.5 (3.9) |  | .063 |
| **WOMAC Post Steifheit** | 2.0 (1.9) | 2.0 (1.8) |  | .933 |
| **WOMAC Post Funktion** | 14.8 (14.0) | 14.6 (13.8) |  | .843 |
| **OA Responder** | 150 (92.6) n=162 | 2290 (89.5)  n=2290 | n=2440 (89.7) | .131 |
| **EQ-5D Prä** | 59.1 (20.6) | 65.4 (19.0) |  | **<.001** |
| **EQ-5D VAS Prä** | 44.9 (22.0) | 51.6 (20.6) |  | **<.001** |
| **EQ-5D Post** | 77.5 (19.9) | 81.5 (17.9) |  | **<.001** |
| **EQ-5D VAS Post** | 67.7 (23.2) | 73.0 (20.8) |  | **<.001** |
| **Total Hip Replacements** | | | | |
| **THRs** | **Rheumatic disease**  n =  mean (SD) | **No rheumatic disease**  n =  mean (SD) |  | **p-value** |
| **WOMAC Prä gesamt** | 63.1 (17.0) | 58.7 (16.5) |  | **.002** |
| **WOMAC Prä Schmerz** | 13.3 (4.1) | 12.2 (3.8) |  | **<.001** |
| **WOMAC Prä Steifheit** | 4.7 (2.0) | 4.9 (1.8) |  | .116 |
| **WOMAC Prä Funktion** | 44.9 (12.9) | 41.5 (12.4) |  | **.001** |
| **WOMAC Post gesamt** | 16.4 (19.3) | 15.5 (16.8) |  | .656 |
| **WOMAC Post Schmerz** | 2.8 (3.9) | 2.5 (3.4) |  | .380 |
| **WOMAC Post Steifheit** | 1.6 (1.8) | 1.6 (1.7) |  | .669 |
| **WOMAC Post Funktion** | 12.0 (14.3) | 11.5 (12.6) |  | .713 |
| **OA Responder n (%)** | 71 (93.4) | 1380 (93.2) | 1451 (93.2) | 0.584 |
| **EQ-5D Prä** | 58.7 (20.8) | 65.3 (19.3) |  | **<.001** |
| **EQ-5D VAS Prä** | 43.5 (22.5) | 51.2 (21.0) |  | **<.001** |
| **EQ-5D Post** | 80.5 (19.5) | 84.5 (17.3) |  | **.010** |
| **EQ-5D VAS Post** | 71.5 (22.8) | 76.6 (20.0) |  | **.005** |
| **Total Knee Replacements** | | | | |
| **TKRs** | **Rheumatic disease**  n =  mean (SD) | **No rheumatic disease**  n =  mean (SD) | **Total**  n = | **p-value** |
| **WOMAC Prä gesamt** | 58.8 (16.4) | 57.8 (16.6) |  | .439 |
| **WOMAC Prä Schmerz** | 13.3 (3.5) | 12.6 (3.6) |  | **.003** |
| **WOMAC Prä Steifheit** | 4.8 (1.8) | 4.9 (1.8) |  | .410 |
| **WOMAC Prä Funktion** | 41.1 (12.6) | 40.4 (12.1) |  | .468 |
| **WOMAC Post gesamt** | 23.7 (17.9) | 25.9 (19.4) |  | .179 |
| **WOMAC Post Schmerz** | 4.8 (3.8) | 4.8 (4.2) |  | .937 |
| **WOMAC Post Steifheit** | 2.4 (1.8) | 2.5 (1.8) |  | .316 |
| **WOMAC Post Funktion** | 17.0 (13.3) | 18.8 (14.2) |  | .151 |
| **OA Responder n (%)** | **79 (91.9)** | **910 (84.5)** | **989 (85.0)** | **.039** |
| **EQ-5D Prä** | 60.5 (20.5) | 65.7 (18.6) |  | **<.001** |
| **EQ-5D VAS Prä** | 46.1 (21.5) | 52.2 (20.2) |  | **<.001** |
| **EQ-5D Post** | 75.3 (20.0) | 77.8 (18.0) |  | .078 |
| **EQ-5D VAS Post** | 64.8 (23.2) | 68.5 (20.8) |  | **.024** |

TJR= Total joint replacements, THR = Total hip replacements, TKR = Total knee replacements
